# Prevalence and genotypes of *Enterocytozoon bieneusi* in wildlife in Korea: a public health concern

**DOI:** 10.1186/s13071-019-3427-6

**Published:** 2019-04-08

**Authors:** Said Amer, Sungryong Kim, Jae-Ik Han, Ki-Jeong Na

**Affiliations:** 1 Laboratory of Veterinary Laboratory Medicine and Wildlife Medicine, Veterinary Medical Center and College of Veterinary Medicine, Chungbuk National University, Cheongju, Chungbuk 28644 Republic of Korea; 2 Department of Zoology, Faculty of Science, Kafr El Sheikh University, Kafr El Sheikh, 33516 Egypt; 3 Laboratory of Wildlife Medicine/Diseases, College of Veterinary Medicine, Chonbuk National University, Iksan, Jeonbuk 54596 Republic of Korea; 4 The Wildlife Center of Chungbuk, Cheongju, Chungbuk 28116 Republic of Korea

**Keywords:** *Enterocytozoon bieneusi*, Prevalence, Wildlife, Korean water deer, Raccoon dog, Genotyping, South Korea

## Abstract

**Background:**

*Enterocytozoon bieneusi* is a unicellular microsporidian fungal pathogen that infects a broad range of animal hosts, including wild and domestic animals and humans. The infection burden of this parasite in wild animals in Korea is largely unknown. In this study, the occurrence and genotypes of* E. bieneusi* were investigated in wild animal populations in Korea.

**Methods:**

A total of 157 fecal samples (97 from Korean water deer, 48 from raccoon dogs and 12 from other taxa) were collected from wild animals at five wildlife centers in Korea. Genomic DNA was extracted from the samples and screened by nested-PCR targeting the internal transcribed spacer (ITS) region of rRNA, followed by sequence analysis to determine the genotype(s) of* E. bieneusi*.

**Results:**

The overall prevalence of* E. bieneusi* was 45.2% (71/157), with rates of 53.6% (52/97) in Korean water deer, 35.4% (17/48) in raccoon dogs and 16.7% (2/12) in other taxa. We detected seven ITS genotypes, including one known (genotype D) and six new genotypes (Korea-WL1–Korea-WL6). Phylogenetically, all detected genotypes clustered with counterparts belonging to group 1, which includes isolates from different animal hosts and humans, suggesting their zoonotic potential.

**Conclusions:**

Our survey results indicate that* E. bieneusi* circulates widely in wild animals in Korea. These findings address the role of wildlife as a potential source of microsporidiosis in domestic animals and humans.

## Background

*Enterocytozoon bieneusi* is a cosmopolitan microsporidian that infects a wide range of vertebrate and invertebrate hosts, including humans, domestic animals and wild game [1–6].* Enterocytozoon bieneusi* infection is associated with enteropathy, resulting in diarrhea, malabsorption and occasionally growth impairment, especially in pediatric and immunocompromised individuals [7–12]. In addition, extra-intestinal microsporidian infections due to* E. bieneusi* are frequently reported [13–15]. Fecal-oral transmission is the most common route of infection through spore-contaminated food and/or water [9, 16–18]. Airborne transmission in cases of respiratory infections is still largely controversial [13]. Treatment options for *E. bieneusi* infections are limited, and few drugs have shown anti-microsporidian activity [9, 19].

Microscopic investigations of *E. bieneusi* spores in stool samples are inadequate to discriminate between genotypes owing to a lack of morphological differences. Molecular biology analyses are widely used for the sensitive detection and genotyping of microsporidian species [20, 21]. The genetic structure of the internal transcribed spacer (ITS) region of rRNA shows high diversity among isolates [22], providing the ability to differentiate between host-adapted and zoonotic genotypes [1, 2, 23] as well as the characterization of the geographical distribution of certain genotypes [24].

*Enterocytozoon bieneusi* is commonly detected in different wildlife, either in captive or free-living populations [5, 25–31], with ample range of genotypes of both host-adapted and those lacking host specificity [1, 32]. Thus, wildlife animals are recognized as environmental reservoir for many human and animal infections [32, 33]. In Korea, a few studies have reported the molecular detection of *E. bieneusi* in livestock [34, 35] and in bats [36]; however, the infection burden of this microsporidian fungal pathogen in wildlife is largely unknown. Therefore, in the present study, the prevalence and genotypes of this parasite in wild animal populations in Korea were determined. Fecal samples were collected from wild animals at wildlife centers. Samples were screened for parasite occurrence by nested-PCR targeting the ITS region of rRNA. Genotypes were determined by sequence analysis of the positive samples. The relatedness of the obtained sequences was determined by alignment with reference sequences in GenBank and phylogenetic analysis.

## Methods

### Collection of samples

**Fig. 1:**
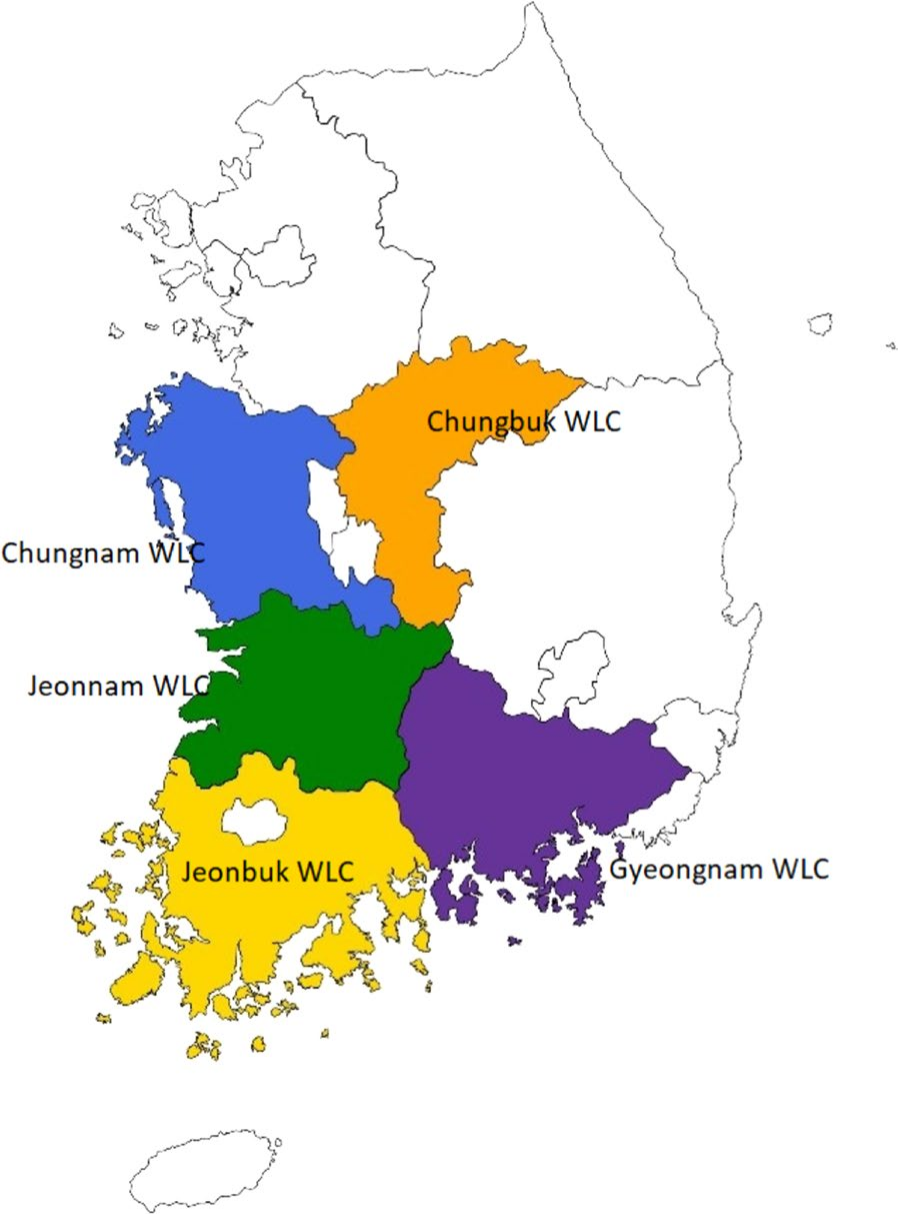
Site map of wildlife centers which contributed to the collection of samples analyzed in the present study

This study was performed from April to September, 2018, using samples collected from wild animals at five Korean wildlife centers (Fig. 1). A total of 157 fecal samples were collected from different animal taxa (Table 1), including 97 samples from Korean water deer (*Hydropotes inermis argyropus*), 48 from raccoon dogs (*Nyctereutes procyonoides*), 2 from roe deer (*Capreolus pygargus*), 3 from leopard cats (*Prionailurus bengalensis*), 3 from Eurasian otters (*Lutra lutra*), 3 from the Siberian weasel (*Mustela sibirica*) and 1 from a Eurasian badger (*Meles meles*). Fresh samples were placed in labeled plastic cups, mixed with 10 volumes of 70% ethanol, and stored at 4 °C. The samples were transported in an ice box to the laboratory of Veterinary Laboratory Medicine at the Veterinary Medical Center, CNBU, Cheongju, Korea.

**Table 1:** Distribution of samples and prevalence of *E. bieneusi* for each wildlife center and animal species

Location	Korean water deer (*n* = 97)		Raccoon dog (*n* = 48)		Other taxa (*n* = 12)		No. infected	Total number	Infection rate (%)
	Infected	Uninfected	Infected	Uninfected	Infected	Uninfected			
Chungbuk	7	11	4	11	0	4	11	37	29.7
Jeonbuk	16	12	13	7	1	2	30	51	58.8
ChungNam	3	6	0	9	1	1	4	20	20.0
JeonNam	3	7	0	4	0	3	3	17	17.7
GyungNam	23	9	0	0	0	0	23	32	71.9
Total	52	45	17	31	2	10	71	157	45.2^a^
Infection rate (%)	53.6		35.4		16.7				

^a^Overall rate

### DNA extraction and PCR amplification

Ethanol was washed off the fecal samples with Milli Q water by centrifugation. Genomic DNA was extracted from 0.2 ml of fecal slurry using a QIAamp DNA Stool Mini Kit (Qiagen, Hilden, Germany) according the manufacturer’s instructions. DNA preparations were screened for *E. bieneusi* by nested PCR as previously described [5], with reagent grade water as a negative control. PCR was performed using 50-µl reaction mixtures consisting of 1× PCR buffer, 200 µM dNTPs, 3 mM MgCl_2_, 260 nM primers, and 1.5 units of *Taq* DNA polymerase (Takara, Tokyo, Japan). Amplified fragments were electrophoresed on a 1.5% gel stained with EcoDye™ and visualized using UV light.

### DNA sequence analysis

Secondary PCR products of positive samples were sequenced in both directions using Big Dye® Terminator v3.1 Cycle Sequencing Kit (Applied Biosystems, Foster City, CA, USA) and an ABI 3130 Genetic Analyzer (Applied Biosystems). Generated sequences were assembled using ChromasPro (https://technelysium.com.au/wp/chromaspro/ v.2.1.8) and aligned with reference sequences in GenBank using ClustalX (http://www.clustal.org/) to determine the occurrence and genotype of *E. bieneusi*. Phylogenetic analysis was performed using maximum likelihood (ML) as implemented in MEGA v.7.0 (https://www.megasoftware.net/) with the Kimura 2-parameter model.

## Results

### Occurrence of* Enterocytozoon bieneusi*

Based on PCR and sequencing, we detected *E. bieneusi* in 71 out of 157 investigated samples, with an overall infection rate of 45.2%. The prevalence was 53.6% (52/97) and 35.4% (17/48) in Korean water deer and raccoon dogs, respectively (Table 1). In addition, one positive sample was detected in each of the roe deer (1/2) and leopard cat (1/3). Samples from other taxa, including three otters, three weasels and one Eurasian badger, were negative. Infection was distributed in animals from all wildlife centers involved in the present study, with the highest infection rate detected at Gyungnam wildlife center (71.9%), followed by Jeonbuk (58.8%), Chungbuk (29.7%), Chungnam (20.0%) and Jeonnam (17.7%).

### *Enterocytozoon bieneusi* genotypes

**Fig. 2:**
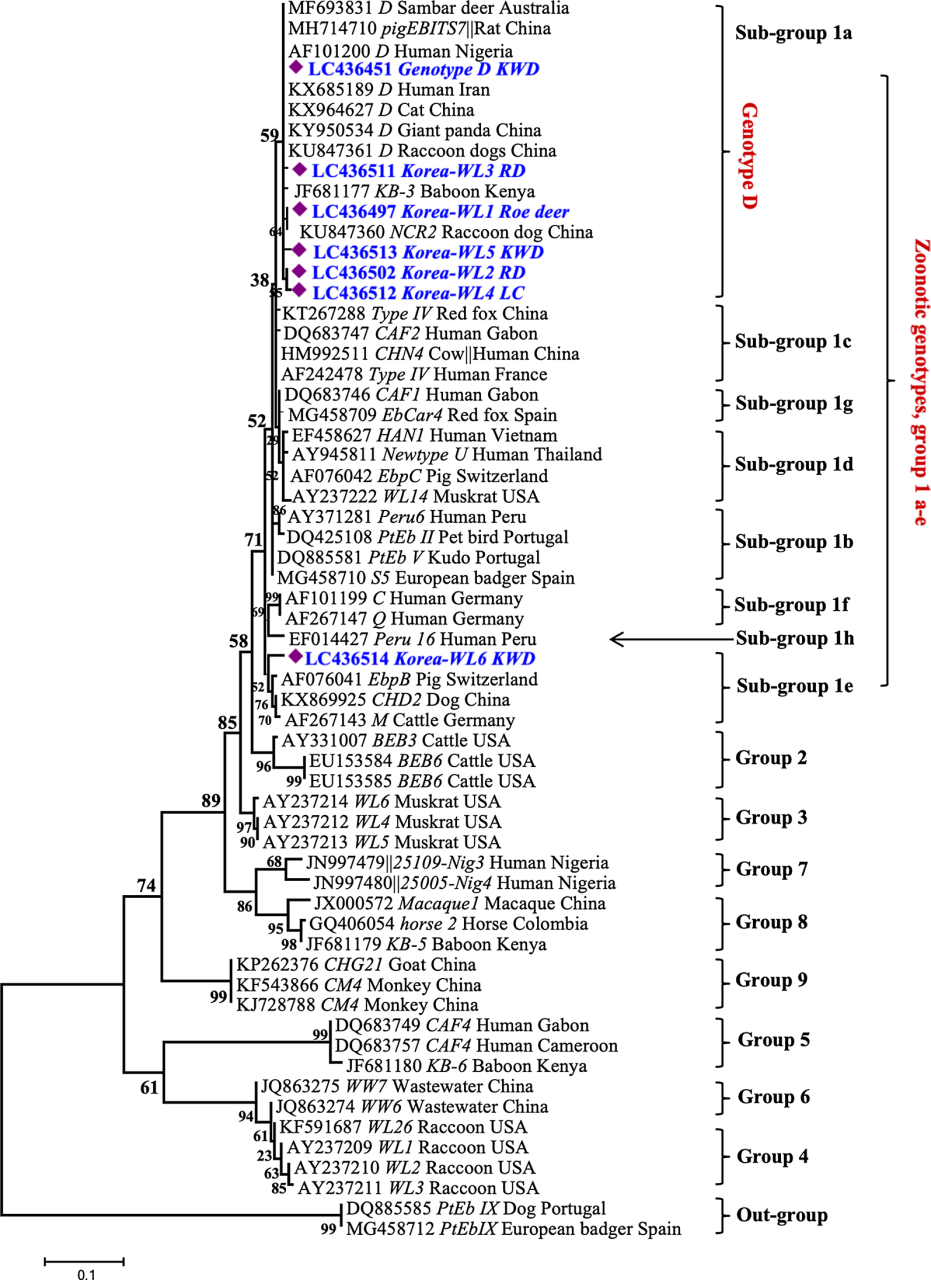
Phylogenetic relationships among *Enterocytozoon bieneusi* from wildlife in Korea and isolates with reference sequences in GenBank. Evolutionary relationships were inferred based on internal transcribed spacer (ITS) sequences using the Maximum Likelihood (ML) method implemented in MEGA7. Branch support on the ML tree was calculated based on 1000 bootstrap replicates. Sequences obtained in this study are marked with diamonds on the tree. In all cases, the branch label includes accession number, followed by genotype name, host animal and country of origin. *Abbreviations*: KWD, Korean water deer; RD, raccoon dog; LC, leopard cat

Based on sequence analysis of the ITS gene marker, we detected seven genotypes, including one known (genotype D) and six novel genotypes (Korea-WL1-WL6). The known genotype D (detected in 35 samples isolated from 29 Korean water deer and 6 raccoon dogs) was identical to KX685189 from a human in Iran [11], KY950543 from a giant panda in China [28] and MF693831 from a sambar deer in Australia [5]. The novel genotypes Korea-WL1 (detected in 21 samples isolated from 12 Korean water deer, 8 raccoon dogs and 1 roe deer), Korea-WL2 genotype (detected in 11 samples isolated from 6 raccoon dogs and 5 Korean water deer) and Korea-WL3 (detected in one sequence isolated from a raccoon dog) showed one nucleotide substitution each at positions 174 (A/G), 277 (T/G) and 232 (A/G), respectively, compared to our genotype D sequences and the reference sequence (MF693831). We detected Korea-WL4 in one sequence (derived from sample of a leopard cat) with two substitutions (A/G and T/G) at positions 152 and 277, respectively. Korea-WL5, also detected in one sequence derived from a sample of Korean water deer, showed a 2-bp deletion at positions 152 and 153 as well as substitutions of T/G and G/A at positions 259 and 298, respectively, compared to genotype D (Table 2). Moreover, Korea-WL6 (generated from sample of a Korean water deer) showed a sequence identity of 98% with KR902354 detected in wastewater in China [37] and KP262358 detected from a goat in China [38], with seven nucleotide substitutions. Phylogenetically, all detected genotypes clustered with counterparts belonging to zoonotic group 1. Genotype D and novel genotypes Korea-WL1-WL5 clustered with other sequences of group 1a, whereas Korea-WL6 clustered with sequences in group 1e (Fig. 2).

**Table 2:** Positions of nucleotide changes within *E. bieneusi* genotype D-related sequences

Genotype/sequence position	152	159	160	174	232	259	277	298	Host animal	Accession no.
Korea genotype D (35 sequences)/MF693831	G	T	G	G	G	G	G	A	KWD, RD	LC436451-LC436482
Korea-WL1 (21 sequences)	.	.	.	A	.	.	.	.	KWD, RD, roe deer	LC436483- LC436501
Korea-WL2 (11 sequences)	.	.	.	.	.	.	T	.	KWD, RD	LC436502-LC436510
Korea-WL3 (1 sequence)	.	.	.	.	A	.	.	.	RD	LC436511
Korea-WL4 (1 sequence)	A	.	.	.	.	.	T	.	LC	LC436512
Korea-WL5 (1 sequence)	.	–	–	.	.	T	.	G	KWD	LC436513

*Abbreviations*: KWD, Korean water deer; RD, raccoon dog; LC, leopard cat; –, deletion

## Discussion

Our results provide the first description of the occurrence and genotypes of the microsporidian *E. bieneusi* in wild animal populations in Korea. We observed an overall prevalence of 45.2%, with the highest infection rate (53.6%) detected in samples from Korean water deer, followed by raccoon dogs (35.4%) and other taxa (16.7%). Little is known about the occurrence of* E. bieneusi* in Korean water deer and free-range raccoon dogs. However, a low infection rate of 4.1% was recently reported in a survey of wild deer in water catchments in Melbourne, Australia [5], with all infections restricted to the sambar deer. The average *E. bieneusi* infection rates are 16.8% in wild reindeers [39], 34.0% in wild Pere David’s deer in China [40] and 32.5% in wild white-tailed deer in the USA [41]. In farmed deer, the prevalence is 7–35% in sika deer, 20–37% in red deer [42–45] and ~17% in musk deer [31] in China. Compared with the 35.4% *E. bieneusi* infection rate in raccoon dogs in our study, a lower prevalence of 4–22% has been reported in farmed raccoon dogs (*N. procyonoides*) in China [29, 46, 47]. Moreover, rates of *E. bieneusi* occurrence differ according to the animal species and geographical region. Danišová et al. [48] reported an infection rate of 1.07% in wild mice in Slovakia, far lower than estimates of 38.9% in different wild rodents in Poland [25]. Additionally, a prevalence of 16.7% was observed in red-bellied tree squirrels in China [27]. A comparable prevalence of 41.2% was detected in Eurasian wild boars in China [28]. Similar rates of infection (~11%) have been observed in the herbivorous giant panda in China [26] and in wild carnivores (**~**13%) in Spain [33]. Humidity and temperature due to precipitation rate and vegetation types in forests in Korea are favorable environmental factors that maintain viability of *E. bieneusi* spores for long time and the social behavior of the Korean water deer and raccoon dogs that facilitates contact among families and groups of animals may be responsible for the high infection rate detected in the present study.

Our PCR and sequence analysis of the ITS region revealed a substantial degree of genetic diversity in the form of nucleotide substitutions and deletions. We identified seven distinct genotypes, including one known (genotype D) and six novel genotypes (Korea-WL1-WL6). Comparable results for genetic diversity and new genotypes have been obtained for isolates from different species of wild and farmed deer in Australia and China [5, 39, 40, 44]. Contrasting our findings, more new genotypes (up to 25) were detected in wild white-tailed deer in the USA [41] and in farmed sika deer in China [45], and only one or two new genotypes were detected in musk deer, red deer and Siberian roe deer in China [31, 43]. Genotype D of *E. bieneusi* detected in raccoon dogs in our study is a common genotype in farmed raccoon dogs in China [46], along with various (3–7) additional known and new genotypes [29, 46, 47]. Results of this study expand the geographical and host range of these genotypes.

Phylogenetic analysis (Fig. 2) revealed that *E. bieneusi* genotypes cluster in roughly eight groups. Genotypes of group 1 have no host specificity and were retrieved from different wildlife, domestic animals and humans, and those belonging to groups 2–8 are almost all host-specific [22, 49, 50]. In this study, all detected genotypes were assigned to *E. bieneusi* group 1; 70 sequences belonged to 1a and one sequence belonged to 1e. Furthermore, isolates of genotype D, Korea-WL1 and Korea-WL2 reported in the present study were obtained from different hosts including Korean water deer, raccoon dogs and roe deer, indicating a lack of host specificity. However, the situation of host specificity for Korea-WL3 to Korea-WL6 genotypes is difficult due to isolation from only one sample. Similarly, most previously reported genotypes detected in wild and farmed deer and raccoon dogs belonged to group 1 [5, 29, 39–41, 45–47]. The transmission of group 1 genotypes between animals and humans is likely, indicating that they have zoonotic potential [32, 51]. The exclusive identification of genotypes belonging to group 1 in this study emphasizes the role of wild populations as a source of environmental contamination; these genotypes are potentially hazardous for domestic animals and human health in Korea.

## Conclusions

Our results provide the first evidence for the presence of *E. bieneusi* in wildlife in Korea. All infected animals carried potentially zoonotic pathogenic genotypes of this microsporidian. This study was limited by the uneven collection of samples from different wildlife centers and animal species, which my lead to biases in the results. Therefore, a larger study covering more geographical regions of Korea with even sample collection is underway to ascertain the role of wildlife as a reservoir for zoonotic gastrointestinal pathogen. The obtained results expand the geographical and host range of the detected genotypes and improve our understanding of the epidemiology of* E. bieneusi* infection in humans and animals in Korea.

## Abbreviations

KWD: Korean water deer (*Hydropotes inermis argyropus*)
ITS: internal transcribed spacer of nuclear ribosomal DNA
